# Evening Chronotype Is Associated with Changes in Eating Behavior, More Sleep Apnea, and Increased Stress Hormones in Short Sleeping Obese Individuals

**DOI:** 10.1371/journal.pone.0056519

**Published:** 2013-03-06

**Authors:** Eliane A. Lucassen, Xiongce Zhao, Kristina I. Rother, Megan S. Mattingly, Amber B. Courville, Lilian de Jonge, Gyorgy Csako, Giovanni Cizza, for the Sleep Extension Study Group

**Affiliations:** 1 Clinical Center, National Institutes of Health, Bethesda, Maryland, United States of America; 2 Intramural Research Program, National Institute of Diabetes and Digestive and Kidney Diseases, National Institutes of Health, Bethesda, Maryland, United States of America; 3 Section on Pediatric Diabetes and Metabolism, National Institutes of Health, Bethesda, Maryland, United States of America; 4 Section on Neuroendocrinology of Obesity, National Institute of Diabetes and Digestive and Kidney Diseases, National Institutes of Health, Bethesda, Maryland, United States of America; 5 Department of Laboratory Medicine, Clinical Center, National Institutes of Health, Bethesda, Maryland, United States of America; University of Pennsylvania School of Medicine, United States of America

## Abstract

**Background:**

Short sleep duration and decreased sleep quality are emerging risk factors for obesity and its associated morbidities. Chronotype, an attribute that reflects individual preferences in the timing of sleep and other behaviors, is a continuum from morningness to eveningness. The importance of chronotype in relation to obesity is mostly unknown. Evening types tend to have unhealthy eating habits and suffer from psychological problems more frequently than Morning types, thus we hypothesized that eveningness may affect health parameters in a cohort of obese individuals reporting sleeping less than 6.5 hours per night.

**Methodology and Principal Findings:**

Baseline data from obese (BMI: 38.5±6.4 kg/m^2^) and short sleeping (5.8±0.8 h/night by actigraphy) participants (*n* = 119) of the *Sleep Extension Study* were analyzed (www.ClinicalTrials.gov, identifier NCT00261898). Assessments included the Horne and Ostberg Morningness-Eveningness questionnaire, a three-day dietary intake diary, a 14-day sleep diary, 14 days of actigraphy, and measurements of sleep apnea. Twenty-four hour urinary free cortisol, 24 h urinary norepinephrine and epinephrine levels, morning plasma ACTH and serum cortisol, fasting glucose and insulin, and lipid parameters were determined. Eveningness was associated with eating later in the day on both working and non-working days. Progression towards eveningness was associated with an increase in BMI, resting heart rate, food portion size, and a decrease in the number of eating occasions and HDL-cholesterol. Evening types had overtly higher 24 h urinary epinephrine and morning plasma ACTH levels, and higher morning resting heart rate than Morning types. In addition, Evening types more often had sleep apnea, independent of BMI or neck circumference.

**Conclusions:**

Eveningness was associated with eating later and a tendency towards fewer and larger meals and lower HDL-cholesterol levels. In addition, Evening types had more sleep apnea and higher stress hormones. Thus, eveningness in obese, chronically sleep-deprived individuals compounds the cardiovascular risk associated with obesity.

## Introduction

The prevalence of obesity, 36% among adults in the US, has reached epidemic proportions [1]. The actual reasons for this increase, in addition to eating more and moving less, are not clear [2]. Therefore, identification of causative factors contributing to the obesity epidemic is of great public health importance. In temporal association with the obesity epidemic, an increasing percentage of the US population, from 20% in 1985 to 25% in 2004, has been reporting sleeping less than six hours per night [3]. Because of the co-occurrence of these two phenomena, we and others hypothesized that chronic sleep deprivation and weight gain may be causally related to each other [3]. Recently, an epidemiological study of approximately 65,000 individuals conducted in Europe demonstrated that, in addition to sleep duration, individual predisposition in sleep timing influences body weight [4].

Individual preference for sleep timing and timing of other behaviors is a stable trait, referred to as one’s chronotype. A large twin study demonstrated that approximately 50% of the chronotype features are heritable [5]. In addition to a genetic component, several demographic and developmental factors such as age, ethnicity, and gender influence the expression of chronotype: eveningness preference reaches a peak around age 20 and then progressively moves towards morningness, with men slightly more evening oriented than women [5], [6]. In 1976, Horne and Ostberg developed a questionnaire to classify individuals based on their preferences for sleep timing [7].

In “modern” regimented societies sleep timing, especially during working days, is influenced by social norms, a phenomenon known as “social jet lag” and we are often forced to be awake against our preferred times. Evening types tend to get less and more shallow, non-refreshing sleep during working days [6], [8]. In addition, Evening types experience anxiety and negative mood, tend to have lower self-esteem, perform worse in school and may be more susceptible to stress [9], [10]. In terms of feeding behavior, eveningness has been associated with lower dietary restraint, less healthful dietary habits, and a tendency for a higher body mass index (BMI) [11], [12].

Circadian rhythms are oscillations with a period of approximately 24 h that are generated in the suprachiasmatic nucleus of the hypothalamus. These endogenous rhythms orchestrate most physiological functions and are synchronized to the environment mainly by sunlight, physical activity, feeding and sleep [13]. The constitutive features of circadian rhythms are best revealed in conditions in which environmental factors are kept invariable. Morning and Evening types at their extreme may be shifted by about two to three hours in circadian oscillations of many bodily functions, including body temperature, melatonin, cortisol, and other hormones secretion [14].

In this study, we examined the relationship of chronotype with sleep, food intake, endocrine and metabolic parameters in a cohort of obese subjects who reported sleeping less than 6.5 h per night.

## Methods

### Study Design and Participants

This analysis is part of the Sleep Extension Study, a prospective, randomized, controlled study of obese (BMI 30–55 kg/m^2^) men and premenopausal women, 18 to 50 years old, reporting sleeping less than 6.5 h per night. Details of this study have been previously reported [15]. A total of 126 participants were randomized in a 2∶1 ratio to the Intervention or the Comparison Group, and the Intervention Group was coached to lengthen sleep and improve sleep hygiene with life-style modifications. Data in this manuscript were obtained at the Screening and the Randomization Visits, thus prior to any behavioral intervention.

A 24 h urine collection was started during the first afternoon of the Overnight Randomization Visit. At 8∶00 the following day, after an overnight fast, blood samples, vital signs, and anthropometric measures were obtained. Participants completed the Pittsburgh Sleep Quality Index, Epworth Sleepiness Scale, and the Horne and Ostberg questionnaire [7], [16], [17]. Sleep apnea recordings were performed for one night [18], [19]. Participants were instructed to keep a sleep diary and to wear a wrist actigraphy monitor for the following 14 days. Out of 126 randomized participants, 119 completed the Horne and Ostberg questionnaire and of these 113 recorded dietary intake for three days.

### Ethics Statement

The study was conducted at the NIH Clinical Center in Bethesda, MD, USA, after approval by the Institutional Review Board of the NIDDK, and is listed in ClinicalTrials.gov (identifier: NCT00261898). Each participant signed a written informed consent form.

### Assessments

#### (A) Chronotype questionnaire

The Horne and Ostberg questionnaire inquires about preferred sleep time and daily performance (score range: 16–86) [7]. It contains 19 questions, such as: “at what time would you like to get up?”, “at what time do you feel tired?”, and “what would be the best time to perform hard physical work?”. Based on their scores, individuals were categorized as being either Morning (score: 50–86) or Evening types (score: 16–49). Chronotype was also analyzed as a continuous variable [20].

#### (B) Sleep measurements

Sleep duration was assessed by a wrist actigraphy monitors (Actiwatch-64, Mini Mitter/Respironics/Philips, Bend, OR) that participants (*n* = 115) wore constantly for recording gross locomotor activity in one-minute epochs [21]. Eighty-nine participants provided information on whether the days were working or non-working. Bedtime and risetime were the first and last 10-min period with less than one “active” epoch, respectively. Bedtime and wake-up time were estimated by the investigator according to sleep diaries. When no diaries were available (n = 16/119), best judgment was used. Actiwatch data were analyzed by Actiware-Sleep version 3.4 software. Sleep efficiency was defined as the percentage of time sleeping out of total time in bed. Mid sleep time was the clock time of the center of the sleep duration period, which was calculated by adding half the sleep duration to the bedtime.

The Pittsburgh Sleep Quality Index and the Epworth Sleepiness Scale are questionnaires that assess sleep quality and daytime sleepiness (score ranges: 0–21 and 0–24 with abnormal thresholds of 5 and 10, respectively) [16], [17].

A portable screening device (Apnea Risk Evaluation System, Advanced Brain Monitoring Inc, Carlsbad, CA) provided an estimate of the respiratory disturbance index (RDI): the number of apneas and hypopneas per hour of sleep [18], [19]. An RDI greater than 5 episodes per hour is considered abnormal. The information on RDI score was available in 100 participants.

#### (C) Anthropometric parameters

Height was measured to the nearest centimeter using a wall-mounted stadiometer (SECA 242, SECA North America East, Hanover, MD) and weight was measured using a stand-on-scale in a hospital gown to the nearest 1/10th of a kg (SR555 SR Scales, SR Instruments, INC, Tonawanda, NY). Neck and waist circumference measurements were done using a non-stretch measuring tape in triplicate to the nearest mm. Neck circumference was measured at the minimal circumference with the head in the Frankfort Horizontal Plane and waist circumference was measured at the uppermost border of the iliac crest.

#### (D) Clinical laboratory analysis

Glucose, total cholesterol, and triglycerides were determined with enzymatic methods high-density lipoprotein cholesterol (HDL-C) and low-density lipoprotein cholesterol (LDL-C) with direct homogeneous methods in fasting serum specimens on an automated analyzer (Dimension Vista 1500, Siemens Health Diagnostics, Deerfield, IL). Plasma ACTH, serum cortisol, and insulin levels were assessed with chemiluminescence immunoassays (Immulite 2500, Siemens). Urinary free cortisol and catecholamines were measured in 24 h collections with liquid chromatography-tandem mass spectrometry, and high-performance liquid chromatography, respectively.

#### (E) Dietary assessment

Participants recorded food intake for three consecutive days, preferably two weekdays and one weekend day. At the Screening Visit, they received written and verbal instructions on recording food intake and were instructed to begin record everything they ate or drank beginning when they woke up on the first day until they woke up then next morning. At the Randomization Visit, dietitians and health technicians reviewed the food records with the participants utilizing three dimensional food models. Information on timing and location of meals was also obtained. The data were analyzed using the Nutrition Data System for Research software (versions 2007–2010, Nutrition Coordinating Center (NCC), University of Minnesota, Minneapolis, MN) [22]. Participants also indicated whether days were school- or working days, which we classified as “working days”, or holidays or travel days, which we classified as “non-working days”. An eating occasion was defined as containing at least 20 kcal and being at least 30 min apart from another eating occasion.

#### (F) Statistical analysis

Normality of variables was examined by Q-Q normality plots. Mean and standard deviation (SD) were calculated for variables with a normal distribution; median and interquartile range were used for variables with a skewed distribution (RDI, plasma ACTH, serum cortisol, HDL-C, LDL-C, triglycerides, insulin, 24 h-urinary norepinephrine, 24 h-urinary epinephrine, urinary free cortisol, and food intake after 20∶00). Logarithmic transformation was applied for skewed variables for further analysis. Morning types were compared to Evening types by independent *t*-tests for continuous variables, and Fisher exact test for categorical variables. Paired *t*-test was used to compare sleep and food intake parameters on working *vs.* non-working days.

Relationships between chronotype score and outcome variables were assessed by linear regression, corrected for gender, age, and anthropometric measures, as appropriates. To assess possible predictors of chronotype score, multivariate forward stepwise analyses (*p*<0.05 to enter, *p*≥0.10 to remove) were generated by including each of the variables that correlated with chronotype score in the univariate analysis at *p*<0.10 (bed- and meal times were not included because they are surrogates for chronotype score). This multivariate model was corrected for age and gender. We tested for the presence of interactions among chronotype score, gender and age. Since we found no interactions, we did not include their product in the model. Analyses were performed using SPSS (version 19, IBM SPSS North America, Chicago, IL).

## Results

### Demographic and Anthropometric Characteristics According to Chronotype

Approximately two-thirds of participants were Morning types and they were about three years older than Evening types (Table 1). There were no gender or racial differences between chronotypes. Morning and Evening chronotypes also had similar anthropometrics but the resting heart rate was significantly lower in Morning types. The chronotype score for the whole sample was 53.7±9.9, mean±SD; N = 119 (Men: 52.9±9.7; *n* = 27; Women: 54.0±10.0; *n* = 92; *p* = 0.630).

**Table 1:** Demographic and anthropometric characteristics of study participants.

	Morning chronotype(score 50–86) *n = *80	Evening chronotype(score 16–49) *n* = 39	*p*-value
Chronotype score (range 16–86)	59.1±6.8	42.7±5.0	**<0.001**
Age (years)	41.7±5.9	38.6±7.8	**0.019**
Female	76%	80%	0.692
African-American/White/Other races (*n*)	54/38/9	72/28/0	0.054*0.219**
BMI (kg/m^2^)	38.2±6.3	39.1±6.6	0.470
Waist circumference (cm)	113.0±13.6	114.7±11.5	0.510
Neck circumference (cm)	38.8±3.8	39.6±3.8	0.340
Morning resting heart rate (beats/min)	68.4±10.1	74.0±10.1	**0.007**

Values are means ± SD,

# median and interquartile range for skewed variables, or %. Skewed variables were log-transformed prior to analysis. *P*-values for *t*-tests or *Fisher exact test are depicted and significant *p*-values (<0.05) are bolded.

* Fisher exact test including all races;

** Fisher exact test excluding “Other races”.

### Sleep Characteristics

As expected for short sleepers, 29% of the participants experienced excessive daytime sleepiness and 84% experienced abnormal sleep quality, as assessed by the Epworth Sleepiness Scale and the Pittsburgh Sleep Quality Index, respectively (Table 2). There were, however, no chronotype differences in sleep quality and sleepiness.

**Table 2:** Sleep characteristics of study participants.

	Morning chronotype(score 50–86)*n = *80	Evening chronotype(score 16–49)*n* = 39	*p*-value
Pittsburgh Sleep Quality Index score	8.0±2.7	8.6±2.9	0.263
Epworth Sleepiness Scale score	8.4±4.5	7.6±4.5	0.387
*Self-reported sleep duration by diary (min)*			
Working days	361±56^1^	403±51	**0.002**
Non-working days	420±63	421±39	0.947
*Sleep duration by actigraphy (min)*			
Working days	339±52^1^	346±64	0.576
Non-working days	386±61^1^	379±60	0.625
*Sleep efficiency by actigraphy (%)*			
Working days	81.5±5.7	81.3±8.2	0.914
Non-working days	81.1±7.5	81.0±7.8	0.964
*Sleep apnea measurements*			
RDI (events/h)#	5 (2–12)	10 (7–16)	**0.018**
RDI >5 (events/h)	47%	81%	**0.001** *

Values are means ± SD,

# median and interquartile range for skewed variables, or %. Skewed variables were log-transformed prior to analysis. *P*-values for *t*-tests or *Fisher exact test are depicted and significant *p*-values (<0.05) are bolded.

* Fisher exact test including all races;

** Fisher exact test excluding “Other races”.

1 Sleep duration differed on working *vs.* non-working days in Morning (diary and actigraphy: *p*<0.001) and in Evening chronotypes (diary: *p* = 0.053; actigraphy: *p* = 0.005).

By self-reported diaries, Morning types reported sleeping significantly less than Evening types on working days, a difference that disappeared during non-working days (Table 2). In contrast, sleep duration and sleep efficiency by actigraphy were similar between types, both on working and non-working days. As expected, both types slept longer during non-working *vs.* working days, according to either sleep diary or actigraphy measures. Sleep apnea was about two times less prevalent in Morning types than in Evening types.

**Figure 1:**
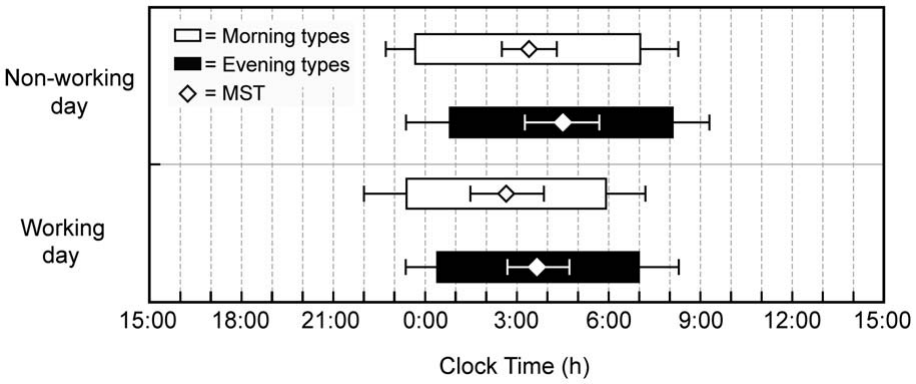
Sleep characteristics per chronotype. Horizontal bars represent sleep time as measured by actigraphy. Bedtime (mean±SD) and risetime (mean±SD) on all days combined and separately on non-working days and on work days in Morning (grey bars) and Evening types (black bars). The mid-sleep times (MSTs) (mean±SD) are depicted by open diamonds and compared between chronotypes by *t*-tests.

Fig. 1 depicts chronotype sleep timing by actigraphy. Both on working and non-working days, Morning types went to bed earlier than Evening types and rose earlier. On non-working *vs.* working days, Morning types went to bed at the same time but rose 49 min later (p<0.001), whereas Evening types went to bed 29 min later (p<0.019) and rose 53 min later (p<0.001).

### Food Parameters

**Figure 2:**
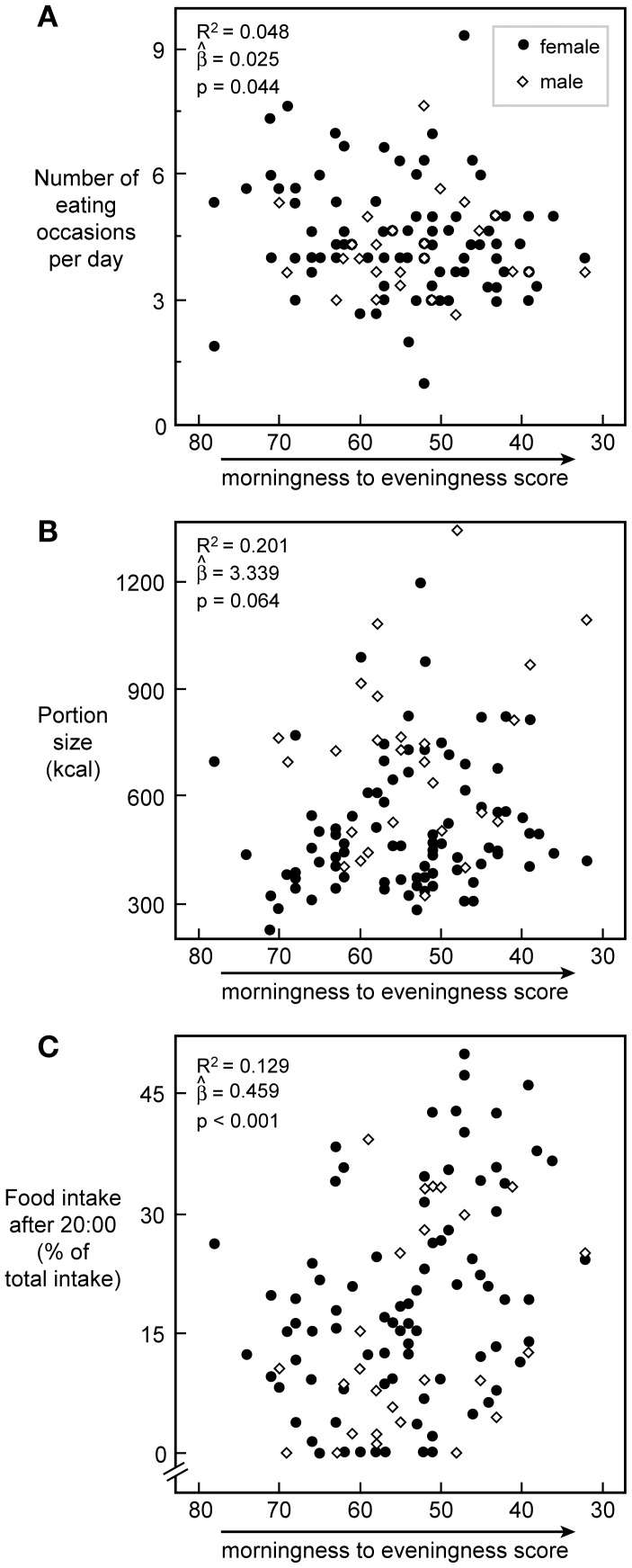
Pattern and timing of food intake *vs.* chronotype score. Plot of chronotype scores versus the number of eating occasions (A), the portion size (B), and the amount of caloric intake after 20∶00 (C) with a trend line, respectively. N = 113 for all. R^2^, slope, and *p*-value for the prediction of chronotype score on food intake variables in each plot are calculated from gender-corrected models. Closed circles represent women, open diamonds represent men.

When examining the three day food recall diary, no differences between chronotypes were observed in total energy intake, portion size, or number of eating occasions, albeit during working days Morning types tended to have smaller portions and to eat more frequently than Evening types (Table 3). During working days, Morning types ate breakfast approximately 1 h and 20 min earlier than Evening types, and after 20∶00 they ate approximately 50% fewer calories in fewer eating occasions. A similar pattern was observed during non-working days. Similarly, as chronotype score moved from morningness towards eveningness, participants had fewer eating occasions, larger portions and consumed more calories after 20∶00 (Fig. 2). Meals consumed after 20∶00 contained less carbohydrate (49±16% *vs.* 53±10%; *p* = 0.021) and protein (12±7% *vs.*14±4%; *p* = 0.006), and tended to contain more fat (34±14% *vs.*32±7%; *p* = 0.069) than meals consumed before 20∶00. There were no differences however between chronotypes in the total amount of fat (*p* = 0.14); carbohydrates (*p* = 0.84); or protein (*p* = 0.89) consumed before and after 20∶00.

**Table 3:** Food intake parameters of study participants divided by chronotype on working *vs.* non-working days.

	Working day			Non-working day		
	Morning type	Evening type	*p*-value	Morning type	Evening type	*p*-value
Total food intake (kcal)	2129±631	2276±815	0.373	2383±928	2378±883	0.922
Portion size (kcal)	461±177	545±219	0.065	599±273	622±380	0.751
Eating occasions per day (*n*)	4.9±1.5	4.4±1.5	0.175	4.2±1.2	4.3±1.6	0.568
First eating occasion (h:min)	7∶17±1∶31	8∶38±1∶52	**<0.001**	8∶56±2∶30	9∶59±2∶32	0.075
Food intake after 20∶00 (kcal)	299±354	677±460	**<0.001**	327±354	537±480	**0.028**
Food intake after 20∶00 (% of total caloric intake)	14±15	30±18	**<0.001**	14±15	24±20	**0.009**

Values are means ± SD. Intake parameters of chronotypes were compared with *t*-tests on working and non-working days separately. Significant *p*-values (<0.05) are bolded.

### Relationship between Chronotype and Anthropometric and Lipid Parameters

**Figure 3:**
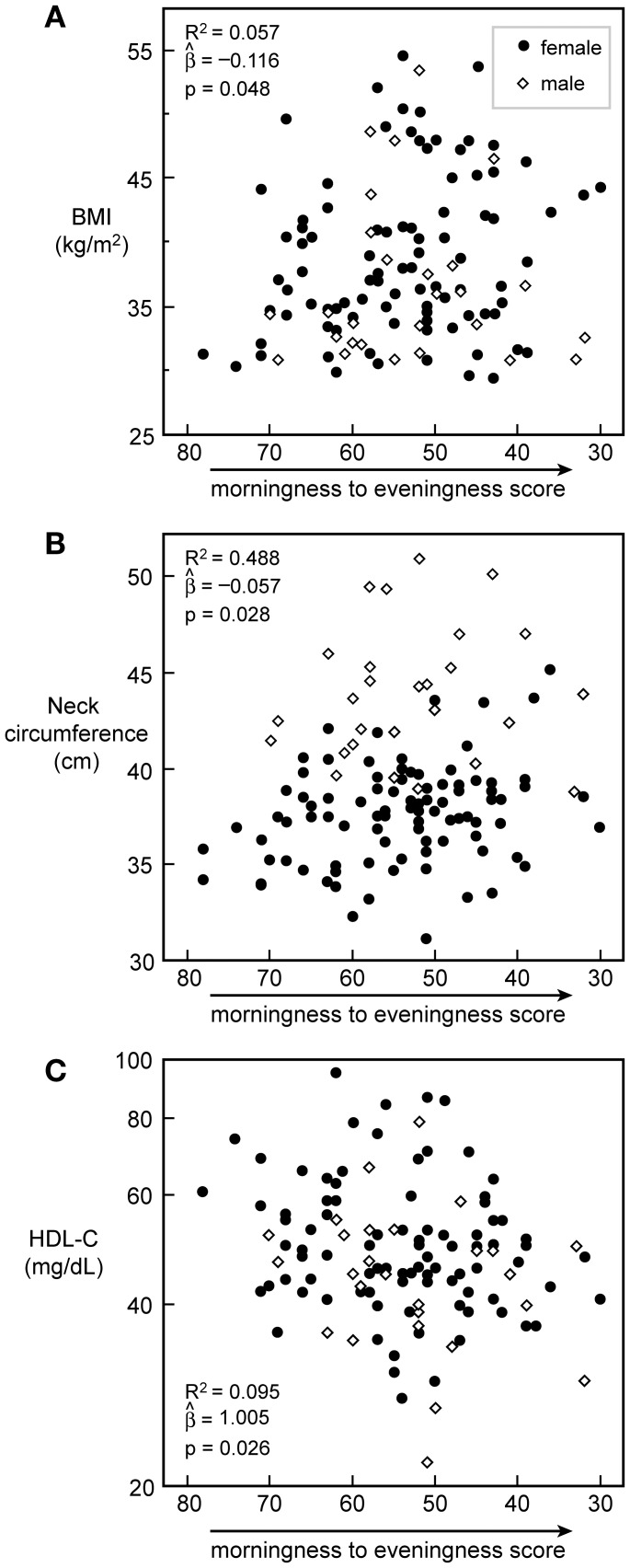
Anthropometrics and HDL-C *vs.* chronotype score. Plot of Chronotype scores versus BMI (A), neck circumference (B), and HDL-C (C) with a trend line, respectively. N = 119 for all. R^2^ slope, and *p*-value for the prediction of chronotype score on variables in each plot are calculated from gender-corrected models. Closed circles represent women, open diamonds represent men.

Moving from morningness towards eveningness scores was associated with an increase in BMI, larger neck circumference, and lower HDL-C levels (Fig. 3). The effect size was remarkable. For example, a 10-unit change in chronotype score was associated with a change of 1.2 kg/m^2^ in BMI (or 3.8 kg in weight), a 0.6 cm change in neck circumference, and a 2.4 mg/dL change in HDL-C levels. Because gender, as expected, was associated with differences in anthropometric measures and serum lipids, all correlations were corrected for gender.

### Endocrine and Metabolic Features

Morning types had higher plasma ACTH and 24 h-urinary epinephrine concentrations and tended to have higher levels of 24 h urinary norepinephrine than Evening types (Table 4). Morning types had also lower resting heart rates than Evening types. A gender-corrected model elicited similar results.

**Table 4:** Endocrine and metabolic parameters of study participants.

	Morning chronotype(score 50–86)*n* = 80	Evening chronotype(score 16–49)*n* = 39	*p*-value
*Endocrine features*			
Morning plasma ACTH (pg/ml)^#^	17 (12–24)	21 (16–32)	**0.019**
Morning serum cortisol (µg/dL)^#^	9 (6–12)	10 (6–14)	0.530
Urinary free cortisol (µg/24 h)^#^	17 (12–24)	19 (10–28)	0.885
Urinary norepinephrine (µg/24 h)^#^	39 (28–56)	45 (37–61)	0.052
Urinary epinephrine (µg/24 h)^#^	3 (2–5)	4 (3–7)	**0.039**
*Metabolic features*			
Insulin (mU/l)^#,^ $	9 (7–14)	10 (6–16)	0.674
Glucose (mg/dL)$	88.6±10.5	90.1±9.6	0.465
Total cholesterol (mg/dL)$	178.9±36.7	177.1±35.5	0.806
Triglycerides (mg/dL)^#,^ $	91 (62–133)	75 (55–106)	0.214
HDL-C (mg/dL)^#,^ $	48 (42–58)	49 (41–52)	0.513
LDL-C (mg/dL)^#,^ $	106 (91–125)	116 (89–134)	0.532

Values are means ± SD or ^#^median and interquartile range for skewed variables.

$ Measured in fasting morning plasma samples. *P*-values for *t*-tests are depicted.

**Figure 4:**
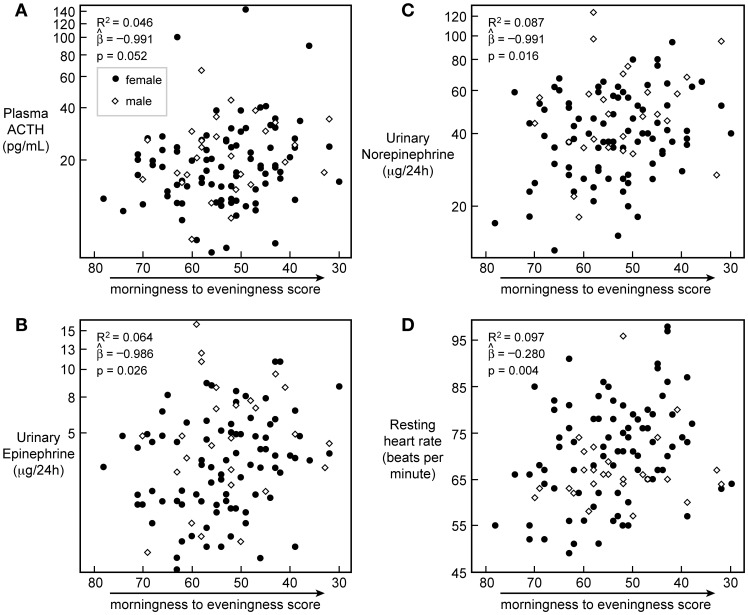
Stress hormones and heart rate *vs.* chronotype score. Chronotype scores on the horizontal axis are regressed against plasma ACTH (*n* = 118; A), 24 h urinary epinephrine (*n* = 110; B) and norepinephrine (*n* = 114; C), and resting heart rate (*n* = 113; D). R^2^ for the gender-corrected model is reported, and slope and *p*-value for the prediction of chronotype score on variables in the model are given. Closed circles represent women, open diamonds represent men.

Progression towards eveningness was associated with higher plasma ACTH levels, higher 24 h-urinary epinephrine and norepinephrine levels, and a higher heart rate (Fig. 4).

### Statistical Modeling

RDI was directly related to plasma ACTH (regression coefficient = 0.132; *p* = 0.009) and 24 h urinary norepinephrine (coefficient = 0.112; *p* = 0.004). RDI remained inversely related to chronotype score after correction for BMI (coefficient = −0.011, *p* = 0.018) and neck circumference (coefficient = −0.009, *p* = 0.037). After correction for BMI, chronotype score remained inversely related to the first eating occasion (coefficient = −0.059, *p*<0.001), caloric intake after 20∶00 (coefficient = −10.687, *p* = 0.001), percent caloric intake after 20∶00 (coefficient = −0.445, *p* = 0.001), and HDL-C concentrations (coefficient = −0.002, *p* = 0.035), portion size (coefficient = 0.025, *p* = 0.052), and eating occasions (coefficient = −3.554, *p* = 0.079).

A gender- and age-corrected multivariate forward stepwise model was fitted with predictors that related to chronotype score at *p*<0.10 in gender-corrected univariate regressions: sleep duration by diary on working days, RDI, BMI, neck circumference, number of eating occasions, portion size, heart rate, HDL-C, plasma ACTH, 24 h urinary epinephrine and 24 h urinary norepinephrine. The following variables did not relate to chronotype score at *p*<0.10: Pittsburgh Sleep Quality Index, Epworth Sleepiness Scale, sleep duration by diary on non-working days, sleep duration and sleep efficiency by actigraphy on working- and non-working days, total food intake, waist circumference, insulin, glucose, total cholesterol, triglycerides, LDL-C, and serum cortisol and urinary free cortisol). A level of significance of *p*<0.05 was required to enter the model (Table 5).

**Table 5:** Multivariate model.

	Step 0 model:Gender+Age	Step 1 model:Gender+Age+Urinary norepinephrine	Step 2 model:Gender+Age+Urinary norepinephrine+RDI
**Model for predicting chronotype score**	R^2^ = 0.019, *p* = 0.578	R^2^ = 0.259, *p* = **0.001**	R^2^ = 0.339, *p* **<0.001**
Gender	β = −0.038, *p = *0.773	β = 0.021, *p = *0.856	β = 0.072,*p = *0.530
Age	β = 0.137,*p* = 0.306	β = 0.273,*p = * **0.028**	β = 0.302,*p = * **0.012**
Urinary norepinephrine (µg/24 h)	N.A.	β = −0.531,*p* **<0.001**	β = −0.512,*p<* **0.001**
RDI (events/hour)	N.A.	N.A.	β = −0.290, *p = * **0.013**

This model depicts a gender- and age-corrected model, generated by multivariate forward stepwise analysis. R^2^ and *p*-value for the models are given, and *p*-values and regression β coefficients for separate variables predicting chronotype score are shown. Significant *p*-values (<0.05) are bolded.

At Step 1, urinary 24 h norepinephrine entered the model and, together with age and gender, accounted for approximately 26% of the variability in chronotype score. At Step 2, the addition of RDI increased the variability in chronotype score accounted for by the model to 34%. Thus, the resulting final model retained two significant predictors, 24 h urinary norepinephrine and RDI, which were both strongly related in an inverse fashion to chronotype score.

## Discussion

In this cohort of sleep-deprived obese subjects, eveningness was associated with an unhealthy eating pattern, characterized by eating later on both working and non-working days and a trend towards a decreased number of eating occasions with larger portion sizes. Evening types were also more likely to have sleep apnea than Morning types, had higher levels of stress hormones, and had a higher resting heart rate. In addition, moving from morningness toward eveningness scores was associated with an increase in BMI and a decrease in HDL-C.

Eating less frequently has been reported to be associated with higher BMI and increased risk of weight gain [23]. Two- *vs.* three-daily meals were less effective in decreasing satiety levels and in inducing fat oxidation in lean women under isocaloric conditions [24]. Similarly, when lean men used to consume four-daily meals were switched to eating three-daily meals for four weeks, less fat was oxidized and fat mass increased [25]. Consistent with our observations, evening types consumed twice as many calories after 20∶00, and had a higher BMI than control subjects, while not differing in total food dietary [26]. In a cross-over, non-randomized study of weight loss, obese women lost more weight and had greater fat oxidation when they were assigned to consume 2/3 of their total caloric intake in the morning *vs.* the evening. However the evening pattern was associated with a larger loss of fat *vs.* fat free mass and there was an order effect that may have influenced the conclusion [27]. A dietary intake study reported that food intake in the late night was less satiating and resulted in greater total energy intake [28]. Interestingly, meals consumed after 20∶00 in our study contained less protein, which may decrease the satiating effect of food, as proteins are thought to be more satiating than carbohydrate [29]. Our results are also in agreement with a Finnish study of approximately 4500 adult subjects with a mean BMI of 27 kg/m^2^ reporting unhealthy dietary habits in Evening types [30].

In our sample, eveningness was associated with lower HDL-C levels, and a trend towards higher BMI and neck circumference. In a study of lean participants, Evening types tended to have increased BMI [12]. Social jetlag (discrepancy between sleep times on working *vs.* non-working days) was directly related to BMI in overweight and obese individuals [4]. Interestingly, overweight/obese individuals carrying the 3111C polymorphism of the CLOCK gene, which is associated with eveningness, were more resistant to weight loss [31].

In our study, in which the actual sleeping schedule was left to the individual subject, we found no differences in sleep quality and sleepiness scores between chronotypes. This is in accord with a well-conducted polysomnography study of 12 Morning types and 12 Evening types, tested in the sleep laboratory according to their habitual sleep schedule in which there were no differences in subjective sleep quality, as well as in total sleep time by EEG [8]. We report here for the first time that sleep apnea was twice as prevalent in the Evening *vs.* the Morning types, independent of BMI and neck circumference. This novel finding has important implications, given the overall negative impact of sleep apnea on health.

In a large population-based, Finnish study, the FINRISK 2007 survey, sleep duration did not differ between chronotypes [32], but Evening types complained more often of insomnia, used sleeping pills more often, and had more frequent nightmares. In addition, the prevalence of short sleepers was significantly greater in men than in women with the Evening chronotype.

In our study sleep duration by actigraphy did not differ between chronotypes either, but Evening types reported sleeping approximately 40 min longer during working days. Further, self-reported sleep duration in both chronotypes was longer than sleep duration by actigraphy and this difference was present on both working and non-working days. We cannot account for the discrepancy between sleep duration by diary and actigraphy. It may take longer for Evening types to fall asleep because they feel less sleepy and may display more active behavior before going to bed. As an internal validation of self-reported measures, participants reported sleeping longer during non-working than during working days. Of note, most of the epidemiological studies on the relationship between sleep and weight relied on self-reported measures [3]. It has been recognized in this field that self-reported sleep and sleep by actigraphy do not correspond closely to each other.

The longer sleep latency period observed in our study for the Evening type may also be due to another related contributory factor, the quality of light. During the waking hours Evening types are less exposed to light, especially in the 100 to 500 lux range, which is the average intensity of interior lights [33]. Light exposure before bedtime delays melatonin onset and may therefore lengthen the period that is needed to fall asleep [34]. Of note, active behavior directly influences the central clock in the hypothalamus and can phase delay it when displayed in the evening hours. This may also lengthen the period that is needed to fall asleep [35]. Under constant routine conditions Evening types are the most alert around 22∶30 whereas Morning types reach their peak of alertness much earlier in the day, around 13∶00 [14].

In our sample, mean sleep efficiency was similarly suboptimal (81%,) in Morning and Evening types. A German actigraphy study conducted in a large sample of young students reported a somewhat better sleep efficiency in Morning types (88%) than Evening types (84%). It is possible that we have observed a “floor” effect in our sample that may have zeroed and obscured chronotype differences in sleep efficiency.

The intriguing possibility that assortative mating, defined as the nonrandom mating of individuals with respect to phenotype and cultural factors, may play a role in chronotype. This has been proposed in a report of a positive relationship in a German sample of 84 couples [36]. Assortative mating, whether positive or not, has been recently listed as one of the 10 putative factors contributing to the obesity epidemic [37]. As Evening types tend to be heavier and this is often already evident at reproductive age, future research should determine whether assortative mating for either factor has additive *vs.* synergistic effects in the etiology of obesity.

Compared to Morning types, Evening types had 20–30% higher 24 h urinary levels of epinephrine and higher morning plasma ACTH levels, indicating an activation of the sympatho-adrenal system. As further evidence, the 24 h urinary norepinephrine was also borderline significantly elevated. In agreement with a previous report [11], resting heart rate was higher in Evening *vs.* Morning types. Evening types have altered heart rate variability and a reduced ability to cope with stress [38]. The fact that ACTH levels were associated with chronotype, points to alterations in the feedback mechanisms of the hypothalamic-pituitary-adrenal system, which has been reported in patients with sleep apnea and obesity [39], [40]. Sleep apnea increases urinary norepinephrine and plasma ACTH levels [40], [41]. However, the trend for increased urinary norepinephrine levels in Evening types was independent from increased sleep apnea. In turn, the chronic hyperventilation syndrome, that is breathing too deeply and too rapidly, as well as spending more time in lighter sleep stages, both more prevalent in Evening than Morning types, are risk factors for sleep apnea [42], [43].

The mean chronotype score, 53.7 seemed to be slightly skewed towards eveningness; for example, a study of 2,526 New-Zealand adults of averagely 40 years of age reported a mean chronotype score of 58.1 [44], and a study of 526 French adults, average age 51, reported a mean score of 59.6 [45]. It is not clear whether cultural and ethnic factors are at play here or whether this represents normal variability.

As indicated by twin studies, there is a clear genetic component to morningness-eveningness, which accounts for more than 50% of its total variance [46]. Albeit subjects tend to become more morning-oriented with age, the age component of the total variance is smaller than the genetic component [46]. As we have recently reviewed, electrification and exposure to artificial light have limited the opportunities of the natural, bright light to synchronize our internal clock to the 24 h day cycle [47]. The magnitude of the synchronizing effect of bright light is dependent on the internal clock as well [48].

Some study limitations should be noted. The cross-sectional nature of this report did not allow establishment of causality. Hormone determinations at a single time-point may have missed endocrine differences between chronotypes in circadian rhythms. Endocrine rhythms shift by two to three hours depending on chronotype [7]. ACTH, for example, has its acrophase around 10∶00 and, since we typically obtained blood at 08∶00, measurements in Morning types were potentially closer to the acrophase [49]. Another limitation of this ancillary study is that the chronotypes were unevenly distributed, with 2/3 of subjects belonging to the Morning chronotype and 1/3 to the Evening chronotype. Finally, we did not perform polysomnography measurements.

In summary, in our sample of obese, sleep-deprived subjects, eveningness was associated with a trend towards higher stress hormones, higher resting heart rate, more sleep apnea, lower HDL-C levels, and a trend towards higher BMI. High BMI and low HDL-C levels predict cardiovascular morbidity [50]. Our findings have public health relevance for the large number of obese individuals who have social jetlag and are sleep-deprived. We advocate for a heightened awareness in the medical field, as well as in the society at large, of the importance of chronotype and of the negative consequences of “forcing” our internal clock. The clinical approach to obese individuals should take into account individual sleep habits and chronotype. A similar characterization should be performed in the industrial sectors that employ shift workers. Finally, controlled studies are needed to test the effects of exposure to bright light on behavior, endocrine and metabolic features in different chronotypes.
